# Genomic insights into prenatal diagnosis of congenital heart defects: value of CNV-seq and WES in clinical practice

**DOI:** 10.3389/fgene.2024.1448383

**Published:** 2024-08-14

**Authors:** Shiyu Sun, Yizhen Ji, Di Shao, Yasong Xu, Xiaomei Yang, Li Sun, Nan Li, Hui Huang, Qichang Wu

**Affiliations:** ^1^ Prenatal Diagnosis Center, Women and Children’s Hospital, School of Medicine, Xiamen University, Xiamen, China; ^2^ BGI Genomics, Shenzhen, China

**Keywords:** CHD, prenatal diagnosis, CNV-seq, WES, chromosomal abnormality

## Abstract

This study aimed to assess the efficiency of CNV-seq and WES in detecting genetic cause of congenital heart disease (CHDs) in prenatal diagnoses and to compare CNV detection rate between isolated and non-isolated CHD cases. We conducted a retrospective study of 118 Chinese fetuses diagnosed with CHD by prenatal ultrasound. Participants underwent CNV-seq and, if necessary, WES to detect chromosomal and single nucleotide variations. The overall detection rate for pathogenic or likely pathogenic chromosomal abnormalities was 16.9%, including 7.6% aneuploidies and 9.3% pathogenic/likely pathogenic copy number variations (CNVs), predominantly 22q11.2 deletion syndrome (54.4%). The sensitivity and specificity of CNV-Seq for detecting P/L*p* CNVs were 95% and 100%, respectively. CNV-Seq offered a 6.7% improvement in detecting chromosomal abnormalities over karyotyping. WES further identified significant single nucleotide and small indel variations contributing to CHD in genes such as TMEM67, PLD1, ANKRD11, and PNKP, enhancing diagnostic yield by 14.8% in cases negative for CNVs. Non-isolated CHD cases exhibited higher rates of detectable chromosomal abnormalities compared to isolated cases (32.4% vs. 9.9%, *p* = 0.005), underlining the genetic complexity of these conditions. The combined use of CNV-seq and WES provides a comprehensive approach to prenatal genetic testing for CHDs, unveiling significant genetic cause that could impact clinical management and parental decision-making. This study supports the integration of these advanced genomic technologies in routine prenatal diagnostics to increase detection diagnostic yields of causal genetic variants associated with CHDs.

## Introduction

Congenital heart disease (CHDs) are the most common birth defects and leading cause of mortality in newborns (Hoffman and Kaplan, 2002). The etiology of CHDs is multifactorial and involves a complex interaction between genetic and non-genetic factors (Pierpont et al., 2018). Genetic factors, including copy number variations (CNVs) and single nucleotide variations (SNVs), account for 15%–25% of congenital heart diseases and often lead to complex cardiac anomalies accompanied by extracardiac malformations such as intellectual disability and developmental delay (Blue et al., 2012).

Prenatal diagnosis of genetic abnormalities leading to congenital heart disease holds significant clinical significance (Rychik et al., 2004). Traditional diagnostic techniques, such as karyotyping and fluorescence *in situ* hybridization (FISH), have been the mainstream approaches for identifying chromosomal abnormalities associated with CHDs (Pierpont et al., 2018; van Nisselrooij et al., 2020). However, these techniques have limitations and weaknesses in their ability to detect chromosomal abnormalities with high resolution and coverage (Callaway et al., 2013; Liu et al., 2022).

Emerging technologies, such as chromosomal microarray (CMA) and CNV-seq, are revolutionizing the field of prenatal diagnosis by providing high-resolution detection of genome-wide CNVs (Talkowski et al., 2012). CNV-seq, based on next-generation sequencing (NGS) platforms, offers the potential to identify a wide range of genetic abnormalities, including small-scale deletions and duplications, in a more comprehensive and accurate manner (Talkowski et al., 2012).

In addition to chromosomal aberrations and CNVs, mutations at the single base-pair level also contribute to the genetic causes of CHDs, although they are inherited in a minority of cases and not in a Mendelian pattern (Jin et al., 2017). Whole exome sequencing (WES) has emerged as a robust tool for diagnostic applications, enabling the identification of genetic variants at the exonic level (Jin et al., 2017; Mone et al., 2021).

The combination of CNV-seq and whole exome sequencing provides a comprehensive approach to identify genetic causes of congenital heart disease in fetuses (Chen et al., 2022). In this retrospective study, we aimed to evaluate the clinical application of CNV-Seq and WES in the prenatal diagnosis of CNVs and SNVs in unselected fetuses with CHD. In addition, we compared the potential diagnostic rates for different CHD subgroups to better understand the genetic causes of ultrasound abnormalities and to make recommendations for prenatal genetic testing for each type of ultrasound abnormality.

## Materials and methods

### Participants

In this single-center retrospective study, we conducted a comprehensive evaluation of chromosomal abnormalities in Chinese fetuses with CHD at Xiamen Maternal and Child Health Hospital over a specified period. Sample collection for this study was approved by the Institutional Reviewer Board of Xiamen Women and Children’s Hospital (KY-155-K01). Informed written consent was obtained from every participant. The study included fetuses diagnosed with CHD through prenatal ultrasound, with or without additional structural anomalies or soft markers. Pregnant women who opted for CNV-seq for etiological diagnosis, along with both parents consenting to further WES analysis and providing samples, were included. Exclusion criteria involved fetuses with specific ultrasound findings such as isolated persistent left superior vena cava, valve insufficiency, coronary artery malformation, or heart tumors. Referrals were made to the prenatal diagnostic center for genetic testing and counseling, with invasive prenatal diagnostic procedures conducted for couples volunteering for prenatal CNV-seq diagnosis. Cases with positive CNV-seq results underwent genetic counseling, while WES was performed in cases with negative CNV-seq results. The study adhered to ethical guidelines, with detailed data collection and analysis methods employed to assess the prevalence of chromosomal abnormalities in these Chinese fetus cohorts with CHD.

### Copy number variation sequencing (CNV-Seq)

Fetal samples were obtained from amniocytes or amniotic fluid depending on gestational age. The amniotic fluid and umbilical cord blood samples were cultured and karyotyped according to standard cytogenetic protocols. Nucleic acid extraction kits (MagPure Universal DNA KF, MGBio) were used according to the standard extraction SOP for the extraction of 15 mL amniotic fluid DNA or 2 mL umbilical cord blood in EDTA tube, with quality control conducted on the extracted quality. The total amount of DNA was required to be ≥ 500 ng, and amniotic fluid samples with a concentration <1 ng/ul were subjected to electrophoresis to ensure DNA bands were not degraded. During library construction, DNA starting with a quantity of 50 ng and a total system volume of 20 µL underwent normalization treatment, followed by enzymatic digestion, end repair, adaptor ligation, and purification. Fragment selection was conducted through PCR amplification. The PCR was conducted with an initial denaturation at 95°C for 5 min, followed by 28 cycles of 95°C for 30 s, 58°C for 30 s, and 72°C for 1 min, with a final extension at 72°C for 10 min. The sequencing proceeded after two rounds of cycle quality control, with post-sequencing data quality control: raw data Q30 ≥ 85%.

After sequencing data were obtained and basic quality control was performed, the data, which were single end sequenced with a read length of 35 bp and an average sequencing depth of 0.4X, were analyzed for alignment with the reference genome. The human genome reference sequence version GRCh37 (UCSC Database, http://genome.ucsc.edu/cgi-bin/hgGateway) was selected. Magi et al.'s CNV detection algorithm was applied to analyze the sequencing data, with detected CNVs having a resolution of 100 kb or higher (Magi et al., 2013). Variations were annotated and interpreted using OMIM (GRCh37; https://www.omim.org/), DECIPHER (v11.4; https://www.deciphergenomics.org/), and ClinGen (GRCh37; https://www.ncbi.nlm.nih.gov/projects/dbvar/clingen/index.shtml). Chromosomal abnormalities were confirmed by karyotyping or Quantitative fluorescent PCR (QF-PCR).

### Whole exome sequencing

Fresh blood from subjects was collected in EDTA tube (5 mL) and genomic DNA was extracted according to the instructions of the reagent kit (MagPure Buffy Coat DNA Midi KF Kit, MAGEN). A total of 200 ng of genomic DNA from each sample was physically fragmented into small fragments primarily ranging from 100 to 500 bp using a sonicator (M220 Focused-ultrasonicator, Covaris). The fragmented DNA was then selected with magnetic beads (VAHTS™ DNA Clean Beads, VAZYME), with the main fragment size after selection being 200–300 bp. After end-repairing and adding an “A" base to the 3′end, the DNA fragments were ligated with adapters that contain a “T" base. The ligated products were purified using magnetic beads (VAHTS™ DNA Clean Beads, VAZYME), followed by PCR amplification and purification to complete the library construction for each subject’s sample. The PCR was performed with an initial denaturation at 95°C for 5 min, followed by 26 cycles of 95°C for 30 s, 58°C for 30 s, and 72°C for 1 min, concluding with a final extension at 72°C for 10 min. The whole-exome capture was performed using the Agilent SureSelect kit according to the manufacturer’s protocol. The libraries were assessed for fragment size and concentration using CaliperGX 1000 Kit, and upon passing these quality checks, the libraries were circularized. The circularized libraries were sequenced using the high-throughput sequencing platform MGISEQ-2000, with a sequencing type of PE100 + 10bp. Upon completion of sequencing, raw sequencing data were obtained, with the data not less than 120G.

The bioinformatics analysis process began with the evaluation of sequencing quality using SOAPnuke software to assess raw data from the sequencing platform, removing low-quality and adapter contaminated reads to obtain clean reads. The clean reads were aligned to the hg19 reference genome using BWA software (Burrows Wheeler Aligner), with concurrent assessment of sequence capture efficiency. SNVs and insertions and deletions (InDels) were called using GATK software. These results were then compared against multiple databases (NCBI dbSNP v147, dbNSDP v2.9.1, ESP6500 v2 HapMap, 1000 human genome dataset, and database of 100 Chinese healthy adults) to identify mutations. CNV detection was conducted using CNVkit and annotated by CNVnator, while SV detection was completed using lumpy software. Variants were interpreted for pathogenicity according to ClinVar, OMIM, HGMD, and ACMG guidelines. Detection of genomic dynamic mutations was performed using ExpansionHunter software. For all detected variants, pathogenicity interpretations were conducted according to ACMG guidelines.

For all identified pathogenic mutations, primers were designed upstream and downstream of the mutation site. PCR amplification was conducted, and the products were sequenced using the Sanger method.

### Statistical analysis

Statistical analysis in this study was conducted using R software (version 4.0). The rates of chromosomal abnormalities, aneuploidies, and CNVs were compared among isolated CHD, non-isolated CHD, simple CHD, and complex CHD groups using Fisher’s exact test or the chi-square test depending on the data characteristics and suitability of each test. The incremental yield of exome sequencing was calculated using the formula: (number of pathogenic or likely pathogenic variants)/(total number of cases − cases with pathogenic CNV-Seq findings). Using QF-PCR as the gold standard, the sensitivity and specificity of CNV-Seq for detecting pathogenic or likely pathogenic CNVs were calculated. Statistical significance was determined at a *p*-value threshold of less than 0.05 for all intergroup comparisons.

## Results

### Characteristics of the cohort

Between January 2020 and December 2022, our center conducted invasive prenatal diagnoses on 118 pregnant women suspecting fetal congenital heart defects (CHD). The participating women had an average age of 29.9°years, with ages ranging from 20 to 44 years (Table 1). The invasive procedures occurred at a mean gestational age of 25.5 weeks, ranging from 12 to 30 weeks (Table 1; Supplementary Table S1).

**TABLE 1:** Clinical Characteristics of the study cohort.

	Total (N = 118)
Maternal age	
Mean (SD)	29.9 (4.04)
Range	20–44
Weeks of gestation	23 weeks (12–30)
CHD type	
VSD	22 (18.6%)
TOF	21 (17.8%)
LVOTD	14 (11.9%)
TGA	12 (10.2%)
Septal defect	11 (9.3%)
SV	10 (8.5%)
RVOTD	7 (5.9%)
TA	5 (4.2%)
Others	16 (13.6%)
Isolated CHD	
Yes	81 (68.6%)
No	37 (31.4%)
Complex CHD	
Yes	90 (76.3%)
No	28 (23.7%)
Outcome	
Continue the pregnancy	24 (20.3%)
Selective fetocide	1 (0.8%)
Termination	93 (78.8%)

Abbreviation: CHD, congenital heart disease; LVOTD, left ventricular outflow tract defect; RVOTD, right ventricular outflow tract defect; SV, single ventricle; TA, truncus arteriosus; TGA, d-transposition of great arteries; TOF, tetralogy of Fallot; VSD, ventricular septal defect.

Among the 118 fetuses evaluated, 81 (68.8%) presented with isolated congenital heart diseases (CHDs), whereas 70 (31.4%) exhibited additional complications, including soft markers, fetal growth restrictions, or other structural anomalies (Table 1). Simple CHDs were identified in 28 fetuses (23.7%), while the remaining 90 fetuses (76.3%) were classified as having complex CHDs (Table 1).

According to the anatomical classification proposed by American Heart Association (AHA) Guidelines, the subjects were grouped into nine primary categories. The most five frequent cardiac anomalies identified included ventricular septal defects (VSD, 18.6%), tetralogy of Fallot (TOF, 17.8%), left ventricular outflow tract obstructions (LVOTD, 11.9%), transposition of the great arteries (TGA, 10.2%), and septal defects (9.3%) (Table 1).

## CNV-seq findings in 118 CHD cases

**FIGURE 1:**
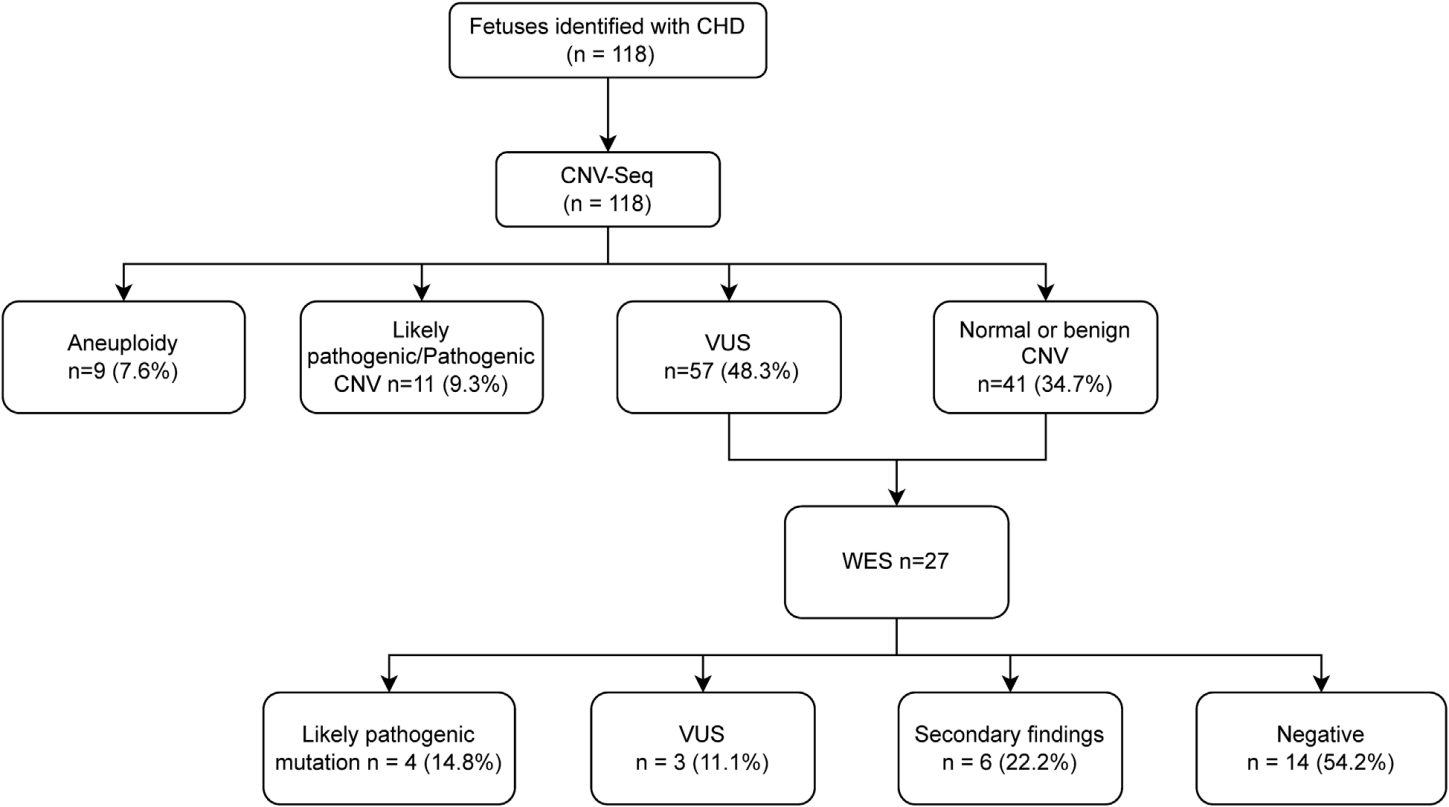
Flow chart of the study and overall results.

The overall detection rate for pathogenic/likely pathogenic (P/L*p*) chromosomal abnormalities was 16.9% (Figure 1). This included 9 cases (7.6%) of aneuploidies, including 5 cases of trisomy 21, two of trisomy 18, and one of trisomy 13 and one of 45,X (Table 2). Pathogenic/likely pathogenic copy number variations (CNVs) were identified in 11 (9.3%) cases, predominantly the 22q11.2 deletion syndrome, representing 54.4% of these CNVs (Table 2). Using QF-PCR as the gold standard, the sensitivity of CNV-SEQ for detecting pathogenic or likely pathogenic CNVs was 95% (95% CI 76%–100%) and the specificity was 100% (95% CI 96%–100%). A comparative analysis revealed that CNV sequencing (CNV-seq) offered a 6.7% improvement in the detection of chromosomal abnormalities over karyotyping (Table 2). Additionally, variants of unknown significance (VUS) were detected in 57 cases (48.3%), while the remaining 41 fetuses displayed normal or benign CNV-seq results (Figure 1).

**TABLE 2:** List of pathogenic CNVs identified by CNV-Seq.

	Karyotype	CNVs	Case number	Size	Known syndrome	Outcome
Aneuploidy						
	Trisomy 21	Trisomy 21	5		Down	Termination
	Trisomy 18	Trisomy 18	2		Edward	Termination
	Trisomy 13	Trisomy 13	1		Patau	Termination
	45, X	45,X	1		Turner	Termination
Total (Aneuploidy)			9			
P/L*p* CNVs						
	46, XN	del 20p12.1p12.3	1	4.56M	Alagille syndrome	Termination
	46, XN	del 22q11.21	1	2.01M	DiGeorge syndrome	Termination
	46, XN	del 22q11.21q11.21	1	2.82M	DiGeorge syndrome	Termination
	46, XN	del 22q11.21q11.21	1	3.00M	DiGeorge syndrome	Termination
	46, XN	del 22q11.21q11.21	1	3.03M	DiGeorge syndrome	Termination
	46, XN	del 22q11.21q11.21	1	3.09M	DiGeorge syndrome	Termination
	46, XN	del Xp21.1p21.1	1	165.36K	-	Termination
	46,XN,dup (1) (q32q41)	dup 1q32.1q41	1	10.37M	-	Termination
	47, XN, +21	dup 22q11.21	1	2.82M	-	Termination
	47,XN,+der (9) (9; 16) (q21; q21)mat	dup 9p24.3q21.13	1	74.03M	-	Termination
	46, XN	del 19p13.3p13.3	1	1.04 Mb	-	Termination
Total (P/L*p* CNVs)			11			

^a^ pathogenic; LP, likely pathogenic.

### Comparative detection rates by CNV-Seq

Table 3 presents the detection rates of CNV-seq among isolated and non-isolated CHD groups. Non-isolated cases exhibited a significantly higher overall detection rate compared to isolated CHD cases (32.4%, 12/37% vs. 9.9%, 8/81, *p* = 0.005) (Table 3). Among them, non-isolated group and isolated group have similar detection rates of P/L*p* CNV (10.8%, 4/37% vs. 8.6%, 7/81, *p* = 0.972) (Table 3). The difference in the detection rate of fetal aneuploidy between the groups was significant (21.6%, 8/37% vs. 1.2%, 1/81, *p* < 0.001) (Table 3).

**TABLE 3:** CNV-Seq results in different types of CHD.

CHD type	Number of fetuses	Aneuploidy (%)	22q11.2 (%)	Other P/LP CNVs(%)	Total (%)
Isolated CHD	81	1 (1.2%)	4 (4.9%)	3 (3.7%)	8 (9.9%)
Non-Isolated CHD	37	8 (21.6%)	1 (2.7%)	3 (8.1%)	12 (32.4%)
Isolated CHD vs. Non-Isolated CHD		<0.001	0.946	0.576	0.005
Total	118	9 (7.6%)	5 (4.2%)	6 (5.1%)	20 (16.9%)

Abbreviations: CHD, congenital heart disease; CNVs, copy number variations; *p*, pathogenic; LP, likely pathogenic.

### Exome sequencing findings

In total, 98 CHD fetuses with negative or VUS results from CNV-seq were recommended for WES. After genetic counseling, 27 cases were recalled and received WES testing after genetic counselling, of which 20 fetuses were sequenced as proband–parent trios and seven fetuses as proband-only. Subsequent analysis detected five sequence variants in 4 cases, enhancing the diagnostic yield of ES testing to 14.8% (4/27) among CHD fetuses without chromosomal aneuploidy or P/L*p* CNV on CNV-seq (Table 4). These variants included one missense mutation, one splicing mutation, and three non-sense mutations, impacting genes such as TMEM67, PLD1, ANKRD11, and PNKP (Table 4). Additionally, VUS were detected in three fetuses (11.1%), all of which were missense variants with autosomal dominant inheritance (Table 4). Secondary findings, as recommended by ACMG, were identified in six cases (22.2%) (Supplementary Table S2).

**TABLE 4:** Details of variants detected by whole exome sequencing (WES) in 27 fetuses with CHD.

*Case*	Gene	Variant	Zygosity	Inheritance	Clinical Classification	CHD	Isolated CHD	Disease or Syndrome
XM_047	TMEM67	c.224G>A (p.Gly75GLu)	Het	AR, paternal	LP	Others	No	OMIM:615991
XM_114	PLD1	c.2334T>A (p.Tyr778*)	Het	AR, paternal	LP	RVOTD	Yes	OMIM:212093
XM_114	PLD1	c.550G>T (p.Glu184*)	Het	AR, maternal	LP	RVOTD	Yes	OMIM:212093
XM_015	ANKRD11	c.988A>T (p.Lys330*)	Het	AD, maternal	LP	RVOTD	Yes	OMIM:148050
XM_019	PNKP	c.199–10_203delCTTTCTGCAGCTGGGinsTCTGAGGGGT	Het	AR, maternal	LP	TGA	Yes	OMIM:605589
XM_042	CDK13	c.869C>T (p.Pro290Leu)	Compound Het	AD, paternal	VUS	RVOTD	Yes	OMIM:617360
XM_046	MAP2K2	c.275G>T (p.Arg92Ile)	Het	AD, de novo	VUS	TOF	Yes	OMIM:615280
XM_096	DSP	c.253G>A (p.Ala85Thr)	Het	AD, paternal	VUS	Others	Yes	OMIM:612908
XM_096	DSP	c.992A>G (p.Gln331Arg)	Compound Het	AD, maternal	VUS	Others	Yes	OMIM:612908

Abbreviations: AD, autosomal dominant; AR, autosomal recessive; CHD, congenital heart disease; Het, heterozygous; LP, likely pathogenic; RVOTD, right ventricular outflow tract defect; TGA, d-transposition of great arteries; TOF, tetralogy of Fallot; VUS, variants of unknown significance.

### Pregnancy outcomes

Perinatal outcomes were available for all 118 cases. Among them, 83.5% (98/118) chose to termination or selective feticide in twins. Among the fetuses diagnosed with chromosomal abnormalities *via* CNV-seq, 85.0% (17/20) chose termination (Supplementary Table S3). All four fetuses with LP gene variants detected by WES also chose termination. There was a significant difference in termination and survival rates between isolated and non-isolated groups (72.8%, 59/81% vs. 27.2%, 22/81% and 94.6%, 35/37% vs. 5.4%, 2/37, *p* < 0.01) (Supplementary Table S3).

## Discussion

This retrospective study at our center conducted prenatal diagnoses on 118 pregnancies suspecting fetal CHD. The cohort’s data indicates a significant prevalence of complex CHDs, with a detection rate of pathogenic or likely pathogenic chromosomal abnormalities at 16.9% using CNV-seq. This rate includes 7.6% of aneuploidies and 9.3% of significant CNVs, primarily the 22q11.2 deletion syndrome. Additionally, the use of WES in cases negative for aneuploidies or pathogenic CNVs *via* CNV-seq further enhanced the diagnostic yield to 14.8%. The findings underscore the effectiveness of CNV-seq combined with WES in enhancing the detection of genetic anomalies in prenatal settings. This improvement is crucial for understanding the genetic causes of CHDs, potentially guiding therapeutic interventions and parental decision-making.

In our study, we observed a 16.9% detection rate of pathogenic or likely pathogenic chromosomal abnormalities in fetuses with CHD using CNV-seq, which presents a noteworthy comparison to other research. For example, Xing et al. reported a lower detection rate, potentially due to their combined use of CMA and exome sequencing (Xing et al., 2022). Conversely, higher detection rates in studies by Lu et al. and Qiao et al. may reflect methodological differences or different inclusion and exclusion criteria of study cohorts (Qiao et al., 2021; Lu et al., 2022). Similar to our study, Mustafa et al. and Zhu et al. acknowledged the efficacy of CMA or CNV-Seq in complex CHD cases, suggesting a consistent recognition of their value across studies (Zhu et al., 2016; Mustafa et al., 2020). The significant variance in detection rates, especially between isolated and non-isolated CHD cases in our study, underscores the influence of genetic diversity and the chosen diagnostic approach, highlighting the importance of integrating multiple genomic technologies to enhance diagnostic accuracy.

In the current study, non-isolated CHD consistently exhibit a higher rate of detectable chromosomal abnormalities compared to isolated cases. This observation is corroborated by several studies including Mustafa et al. (2020); Qiao et al. (2021); Xing et al. (2022) which all highlight the complex genetic landscape in non-isolated CHDs as detected by CMA and exome sequencing. These findings suggest a heightened genetic predisposition to additional anomalies in non-isolated cases, underscoring the necessity for comprehensive genetic evaluations. Clinically, these results advocate for more robust prenatal screening protocols and tailored genetic counseling that considers the increased risk and genetic complexity associated with non-isolated CHDs. Such insights are crucial for optimizing patient management, potentially guiding more precise interventions and informing decisions regarding prenatal care and postnatal outcomes.

In the comparative analysis between CNV-seq combined with WES and CMA with WES for prenatal diagnosis, CNV-seq exhibits several distinct advantages. Leveraging NGS technology, CNV-seq offers a wider detection range, enabling identification of chromosomal abnormalities at lower mosaic ratios and requiring minimal DNA samples (Chen et al., 2022). This broader sensitivity is crucial for detecting clinically significant yet subtle genomic variants that CMA might missed detect. Additionally, the compatibility of CNV-seq with the NGS platforms used for NIPT and WES facilitates the pooling of libraries, which significantly streamlines the diagnostic process and reduces overall costs. These factors not only enhance the efficiency of genetic screening but also improve the cost-effectiveness, making CNV-seq with WES a superior choice for comprehensive prenatal genetic testing.

Copy number variation (CNV) analysis and whole exome sequencing (WES) offer significant benefits over standard ultrasound and conventional cytogenetic analysis in prenatal testing. CNV analysis can detect small chromosomal changes that traditional methods might miss, providing a more detailed genetic profile of the fetus (Ge et al., 2024). WES can identify single nucleotide changes and small genetic alterations within the coding regions, allowing for the diagnosis of many genetic disorders that cytogenetic methods cannot detect (Farwell et al., 2015; Taylor et al., 2015). While ultrasound is crucial for spotting structural anomalies, it cannot detect molecular-level genetic issues. Conventional cytogenetic analysis is good for finding large chromosomal abnormalities but falls short in detecting smaller changes (Shaffer et al., 2012). However, CNV and WES are more expensive, take longer, and may reveal incidental findings that complicate interpretation. Despite these challenges, using CNV and WES in prenatal testing greatly improves the detection of genetic abnormalities, offering a more precise genetic assessment compared to traditional methods.

The study’s strength lies in its comprehensive use of CNV-seq and WES, providing a robust dataset on chromosomal abnormalities in CHD fetuses. However, the limitation of the study includes its focus on a single center, which may not fully capture demographic and genetic variability. Another limitation is the low number of participants undergoing WES, mainly due to its high cost which limits broader access and application in prenatal diagnostics. Additionally, there is considerable hesitancy within both the clinical community and among expectant parents regarding the use of WES for prenatal testing. Concerns typically revolve around the ethical implications, the complexity of interpreting results, and the potential for incidental findings with uncertain clinical relevance. These factors contribute to a reduced adoption of WES in our study, impacting the comprehensiveness and generalizability of our findings. Future studies should aim to expand the cohort size and include multiple centers to enhance the generalizability of the findings.

This study highlights the important role of advanced genomic technologies, especially CNV-seq combined with WES, in prenatal diagnosis of CHDs. The increased detection rate of fetal chromosomal abnormalities, particularly in patients with non-isolated CHD, demonstrates the potential of these techniques to significantly improve diagnostic accuracy and inform clinical decision-making.

## Data Availability

The original contributions presented in the study are publicly available. The dataset has been deposited to CNSA: https://db.cngb.org/cnsa and the accession number is CNP0006049.
