# Supplementary material for: Genomic insights into prenatal diagnosis of congenital heart defects: value of CNV-seq and WES in clinical practice
Source: Front Genet. 2024 Aug 14;15:1448383. doi: 10.3389/fgene.2024.1448383 (PMC11349688; doi:10.3389/fgene.2024.1448383)
Supplement: Supplementary file 1 [file Table1.DOCX]

**Supplementary materials**

Table S1 CNV-seq results and basic clinical characteristics

| ID | Isolated CHD | Maternal age | Complex CHD | CNV result | Outcome |
| --- | --- | --- | --- | --- | --- |
| XM001 | Yes | 36 | Yes | other_pCNVs | Termination |
| XM002 | Yes | 29 | Yes | 22q11.2 | Termination |
| XM003 | No | 32 | No | 22q11.2 | Continue the pregnancy |
| XM004 | Yes | 31 | Yes | 22q11.2 | Termination |
| XM005 | Yes | 32 | Yes | 22q11.2 | Termination |
| XM006 | Yes | 26 | Yes | 22q11.2 | Termination |
| XM007 | Yes | 35 | No | other_pCNVs | Continue the pregnancy |
| XM008 | Yes | 30 | No | other_pCNVs | Continue the pregnancy |
| XM009 | No | 34 | Yes | other_pCNVs | Termination |
| XM010 | No | 30 | Yes | other_pCNVs | Termination |
| XM011 | Yes | 24 | Yes | Normal | Continue the pregnancy |
| XM012 | No | 31 | Yes | Aneuploidy | Termination |
| XM013 | No | 30 | Yes | Aneuploidy | Termination |
| XM014 | No | 37 | Yes | VUS | Termination |
| XM015 | Yes | 21 | Yes | VUS | Termination |
| XM016 | Yes | 25 | Yes | VUS | Termination |
| XM017 | Yes | 39 | Yes | VUS | Termination |
| XM018 | Yes | 31 | Yes | Normal | Termination |
| XM019 | Yes | 30 | Yes | Normal | Termination |
| XM020 | No | 29 | Yes | Normal | Termination |
| XM021 | Yes | 31 | Yes | VUS | Termination |
| XM022 | Yes | 29 | Yes | VUS | Termination |
| XM023 | No | 28 | Yes | VUS | Termination |
| XM024 | No | 27 | No | Aneuploidy | Termination |
| XM025 | No | 30 | No | Aneuploidy | Termination |
| XM026 | No | 35 | Yes | VUS | Termination |
| XM027 | Yes | 31 | Yes | Normal | Termination |
| XM028 | Yes | 28 | No | Normal | Continue the pregnancy |
| XM029 | No | 30 | No | other_pCNVs | Termination |
| XM030 | No | 35 | No | Normal | Continue the pregnancy |
| XM031 | No | 31 | No | Aneuploidy | Termination |
| XM032 | No | 29 | Yes | Aneuploidy | Termination |
| XM033 | No | 26 | Yes | Aneuploidy | Termination |
| XM034 | Yes | 33 | No | VUS | Continue the pregnancy |
| XM035 | Yes | 28 | No | VUS | Continue the pregnancy |
| XM036 | Yes | 26 | No | Normal | Continue the pregnancy |
| XM037 | Yes | 28 | Yes | Aneuploidy | Termination |
| XM038 | No | 31 | Yes | Normal | Termination |
| XM039 | No | 30 | Yes | VUS | Termination |
| XM040 | No | 30 | Yes | VUS | Termination |
| XM041 | Yes | 26 | Yes | VUS | Termination |
| XM042 | Yes | 23 | Yes | VUS | Termination |
| XM043 | No | 35 | No | VUS | Termination |
| XM044 | Yes | 20 | Yes | VUS | Termination |
| XM045 | Yes | 33 | Yes | VUS | Termination |
| XM046 | Yes | 43 | Yes | VUS | Termination |
| XM047 | No | 30 | Yes | VUS | Termination |
| XM048 | Yes | 30 | No | VUS | Continue the pregnancy |
| XM049 | Yes | 32 | No | VUS | Continue the pregnancy |
| XM050 | No | 26 | Yes | VUS | Termination |
| XM051 | No | 29 | No | VUS | Termination |
| XM052 | Yes | 26 | Yes | VUS | Termination |
| XM053 | No | 27 | Yes | VUS | Termination |
| XM054 | Yes | 27 | Yes | VUS | Termination |
| XM055 | Yes | 30 | No | VUS | Continue the pregnancy |
| XM056 | Yes | 28 | Yes | VUS | Termination |
| XM057 | Yes | 30 | Yes | VUS | Termination |
| XM058 | No | 30 | Yes | VUS | Termination |
| XM059 | Yes | 29 | Yes | VUS | Termination |
| XM060 | Yes | 25 | Yes | VUS | Termination |
| XM061 | No | 28 | Yes | VUS | Termination |
| XM062 | No | 29 | Yes | VUS | Termination |
| XM063 | Yes | 32 | Yes | VUS | Continue the pregnancy |
| XM064 | No | 31 | Yes | VUS | Termination |
| XM065 | Yes | 25 | Yes | VUS | Termination |
| XM066 | Yes | 28 | Yes | VUS | Continue the pregnancy |
| XM067 | Yes | 29 | Yes | VUS | Termination |
| XM068 | Yes | 28 | No | VUS | Continue the pregnancy |
| XM069 | Yes | 35 | No | VUS | Continue the pregnancy |
| XM070 | Yes | 23 | No | VUS | Continue the pregnancy |
| XM071 | No | 27 | No | VUS | Termination |
| XM072 | Yes | 30 | Yes | VUS | Termination |
| XM073 | Yes | 35 | Yes | VUS | Continue the pregnancy |
| XM074 | Yes | 29 | Yes | VUS | Termination |
| XM075 | Yes | 27 | Yes | VUS | Termination |
| XM076 | No | 31 | Yes | VUS | Termination |
| XM077 | No | 30 | Yes | VUS | Termination |
| XM078 | No | 26 | Yes | VUS | Termination |
| XM079 | Yes | 31 | Yes | VUS | Termination |
| XM080 | Yes | 28 | Yes | VUS | Continue the pregnancy |
| XM081 | Yes | 21 | Yes | VUS | Termination |
| XM082 | Yes | 41 | No | VUS | Continue the pregnancy |
| XM083 | No | 37 | Yes | VUS | Termination |
| XM084 | No | 30 | Yes | VUS | Termination |
| XM085 | No | 44 | Yes | Aneuploidy | Termination |
| XM086 | Yes | 33 | Yes | Normal | Termination |
| XM087 | No | 30 | No | Normal | Termination |
| XM088 | Yes | 28 | Yes | Normal | Termination |
| XM089 | Yes | 25 | Yes | VUS | Termination |
| XM090 | Yes | 29 | Yes | Normal | Termination |
| XM091 | Yes | 37 | Yes | Normal | Termination |
| XM092 | Yes | 35 | No | Normal | Termination |
| XM093 | Yes | 32 | No | Normal | Continue the pregnancy |
| XM094 | Yes | 25 | Yes | Normal | Continue the pregnancy |
| XM095 | Yes | 31 | Yes | Normal | Termination |
| XM096 | Yes | 30 | Yes | Normal | Termination |
| XM097 | Yes | 27 | Yes | Normal | Termination |
| XM098 | Yes | 28 | Yes | Normal | Termination |
| XM099 | Yes | 30 | Yes | Normal | Termination |
| XM100 | Yes | 34 | Yes | Normal | Termination |
| XM101 | Yes | 29 | Yes | Normal | Termination |
| XM102 | Yes | 31 | No | Normal | Continue the pregnancy |
| XM103 | Yes | 30 | No | Normal | Continue the pregnancy |
| XM104 | Yes | 27 | Yes | Normal | Termination |
| XM105 | Yes | 28 | Yes | Normal | Termination |
| XM106 | Yes | 32 | Yes | Normal | Termination |
| XM107 | Yes | 33 | Yes | Normal | Termination |
| XM108 | Yes | 30 | Yes | Normal | Termination |
| XM109 | Yes | 26 | Yes | Normal | Termination |
| XM110 | Yes | 29 | Yes | Normal | Termination |
| XM111 | Yes | 21 | Yes | Normal | Termination |
| XM112 | Yes | 29 | No | Normal | Termination |
| XM113 | Yes | 29 | Yes | Normal | Termination |
| XM114 | Yes | 32 | Yes | Normal | Termination |
| XM115 | No | 27 | Yes | Normal | Termination |
| XM116 | Yes | 33 | Yes | Normal | Termination |
| XM117 | Yes | 32 | Yes | Normal | Termination |
| XM118 | Yes | 32 | Yes | Normal | Termination |

Table S2 Secondary findings identified in the study

| Case | Gene | Variant | Zygosity | Inheritance | Clinical Classification | CHD | Isolated CHD | Disease or Syndrome |
| --- | --- | --- | --- | --- | --- | --- | --- | --- |
| XM_097 | *TCTN3* | p.Lys260Glnfs*5 | Het | AR, paternal | LP | RVOTD | Yes | OMIM:614815 |
| XM_116 | *CUL3* | p.Lys457Leufs*3 | Het | AD, de novo | LP | TOF | Yes | OMIM:619239 |
| XM_116 | *FBNI* | p.Arg1840Cys | Het | AD, paternal | LP | TOF | Yes | OMIM:102370 |
| XM_117 | *ATIC* | p.Lys357Glnfs*20 | Het | AR, paternal | LP | TGA | Yes | OMIM:608688 |
| XM_038 | *CEP290* | p.Thr18  16Ilefs*3 | Het | AR, paternal | LP | SV | No | OMIM:615991 |
| XM_086 | *PDHA1* | EX4-EX11E  Dup | Semi-Het | XL, maternal | LP | TGA | Yes | OMIM:312170 |

AD, autosomal dominant; AR, autosomal recessive. Het, heterozygous; TOF, tetralogy of Fallot; TGA, d-transposition of great arteries; RVOTD, right ventricular outflow tract defect; CHD, congenital heart defect; LP, likely pathogenic;

Table S3 Pregnancy outcomes grouped by prenatal diagnosis results, and CHD type

|  | Continue the pregnancy (N=24) | Termination (N=94) | Total (N=118) | p value |
| --- | --- | --- | --- | --- |
| Isolated CHD |  |  |  | 0.006 |
| No | 2 (8.3%) | 35 (37.2%) | 37 (31.4%) |  |
| Yes | 22 (91.7%) | 59 (62.8%) | 81 (68.6%) |  |
| WES result |  |  |  | 0.063 |
| LP | 0 (0.0%) | 4 (4.3%) | 4 (3.4%) |  |
| Negative | 0 (0.0%) | 14 (14.9%) | 14 (11.9%) |  |
| WES not performed | 24 (100.0%) | 67 (71.3%) | 91 (77.1%) |  |
| Secondary findings | 0 (0.0%) | 6 (6.4%) | 6 (5.1%) |  |
| VUS | 0 (0.0%) | 3 (3.2%) | 3 (2.5%) |  |
| CNV-seq result |  |  |  | 0.747 |
| Negative | 8 (33.3%) | 33 (35.1%) | 41 (34.7%) |  |
| P/LP | 3 (12.5%) | 17 (18.1%) | 20 (16.9%) |  |
| VUS | 13 (54.2%) | 44 (46.8%) | 57 (48.3%) |  |
